# New Myzopodidae (Chiroptera) from the Late Paleogene of Egypt: Emended Family Diagnosis and Biogeographic Origins of Noctilionoidea

**DOI:** 10.1371/journal.pone.0086712

**Published:** 2014-02-04

**Authors:** Gregg F. Gunnell, Nancy B. Simmons, Erik R. Seiffert

**Affiliations:** 1 Division of Fossil Primates, Duke University Lemur Center, Durham, North Carolina, United States of America; 2 Department of Mammalogy, Division of Vertebrate Zoology, American Museum of Natural History, New York, New York, United States of America; 3 Department of Anatomical Sciences, Stony Brook University, Stony Brook, New York, United States of America; Raymond M. Alf Museum of Paleontology, United States of America

## Abstract

Myzopodidae is a family of bats today represented by two extant species of the genus *Myzopoda* that are restricted to the island of Madagascar. These bats possess uniquely derived adhesive pads on their thumbs and ankles that they use for clinging to smooth roosting surfaces. Only one fossil myzopodid has been reported previously, a humerus from Pleistocene deposits at Olduvai Gorge in Tanzania that was tentatively referred to the genus *Myzopoda*. Here we describe a new genus and two new species of myzopodids based on dental remains from Paleogene deposits in the Fayum Depression in Egypt, and provide an emended diagnosis for the family Myzopodidae. *Phasmatonycteris phiomensis* n. sp. is represented by four specimens from the early Oligocene Jebel Qatrani Formation and *P. butleri* n. sp. is known from a single specimen from the late Eocene Birket Qarun Formation. Together these specimens extend the temporal range of Myzopodidae by 36+ million years, and the geographic range by nearly 4000 kilometers. The new myzopodids, along with previously described bats from the Fayum and Australia, suggest that eastern Gondwana played a critical role in the origin and diversification of several bats clades notably including the superfamily Noctilionoidea, the majority of which live in the Neotropics today.

## Introduction

Myzopodidae is a small family of insectivorous bats that are today endemic to Madagascar. Two living species are recognized: *Myzopoda aurita*, which was described by Milne-Edwards and Grandidier in 1878 [1], and *M. schliemanni*, which was named nearly 120 years later [2]. Both species are characterized by unique morphological specializations that diagnose the family including large, non-pedicellate suction pads on the thumb and ankle, fusion of the tragus to the pinna, and partial obstruction of the external auditory meatus by a mushroom-shaped process [2]–[3]. Long considered a member of the superfamily Vespertilionoidea [4]–[6], Myzopodidae was transferred to the superfamily Nataloidea by Simmons [3] but subsequent analyses based on extensive molecular data sets indicate that Myzopodidae is actually a basal member of the superfamily Noctilionoidea [7]–[12]. Regardless of whether myzopodids are viewed as basal vespertilionoids or basal noctilionoids, dating analyses unambiguously place the origin of Myzopodidae in the Eocene [7]–[10], [12].

Myzopodids have long been considered intriguing for a variety of reasons including their unusual roosting habits – they use the suction pads on their wrists and ankles to cling to the smooth surfaces of broad leaves such as those of *Ravenala* [13]. In this they are similar to New World Disk-winged bats of the family Thyropteridae, but anatomical and evolutionary analyses concur that the wrist and ankle discs in these two groups evolved convergently [3], [14]–[15]. The modern geographic distribution of Myzopodidae is also of interest since close relatives of myzopodids include lineages endemic to Australia and New Zealand (Mystacinidae) and the Neotropics (Noctilionidae, Furipteridae, Thyropteridae, Mormoopidae, and Phyllostomidae) rather than Africa [7]–[9].

The modern fauna of Madagascar encompasses six orders of mammals and includes both endemic and introduced species [16]. The endemic terrestrial mammals (lemuroid primates, tenrecid afrotherians, euplerid carnivorans, nesomyine muroid rodents) can trace their ancestry to Africa, where sister groups to these Malagasy clades occur today [17]. No definitive fossil lemurs have yet been found on the African mainland but tenrecoids, euplerid carnivoran sister groups and nesomyines are known from Africa [18]–[22].

The extant bat fauna of Madagascar includes 49 species representing seven families (Pteropodidae, Emballonuridae, Hipposideridae, Vespertilionidae, Miniopteridae, and Myzopodidae). Many species have been described quite recently –17 species since 2005, with more being described every year. Endemism in Malagasy bats is quite high, with 37 species (55% of the fauna) known only from Madagascar and nearby islands but not from the mainland. In the extant Malagasy fauna, Myzopodidae is the only endemic family; the other bat families known from Madagascar have many mainland representatives in Africa and Asia.

The fossil record of bats from Madagascar is sparse and includes only eleven late Pleistocene and Holocene species (Table 1) [23]. These include two hipposiderid species known only as fossils [24], seven endemic species (known only from Madagascar, the Seychelles and Comoro Islands today), and one species also known from the African mainland (*Hipposideros commersoni*).

**Table 1:** List of extant and extinct bats currently known from Madagascar.

Family	Genus	Species	Type Locality	Endemic	Fossil known	Fossil Only	Notes & References
Pteropodidae	*Eidolon*	*duprenum*	Nossi Bé	X	X		Fossils from Anjohibe Cave [24] & d’Andrahomana [50]
	*Pteropus*	*rufus*	Madagascar	X	X		Fossils from d’Andrahomana [50]
	*Rousettus*	*madagascar-ensis*	Beforona	X	X		Fossils from Anjohibe Cave [24] & d’Andrahomana [50]
Hipposideridae	*Hipposideros*	*commersoni*	Fort Dauphin	X	X		Fossils from Anjohibe Cave [24], Tsimanampetsotsa [51] & d’Andrahomana [50]
	*Hipposideros*	*besaoka*	Anjohibe Cave	X		X	[24]
	*Paratriaenops*	*auritus*	Diégo-Suarez	X			[52]
	*Paratriaenops*	*furculus*	Tuléar		X		Fossils from ?Anjohibe Cave [24], Tsimanampetsotsa [51] & d’Andrahomana [50]
	*Triaenops*	*menamena*	Province de Mahajanga	X			[53]
	*Triaenops*	*rufus*	Eastern Madagascar	X			[54]
	*Triaenops*	*goodmani*	Anjohibe Cave	X		X	[24]
Emballonuridae	*Taphozous*	*mauritianus*	Non-Malagasy				[55]
	*Coleura*	*kibomalandy*	Province d’Antsiranana	X			[56]
	*Paraemballonura*	*tiavato*	Province d’Antsiranana	X			[57]
	*Paraemballonura*	*atrata*	Interior Madagascar	X	X		Fossils from Tsimanampetsotsa [51]
Nycteridae	*Nycteris*	*madagascar-iensis*	North of Ankarana	X			[58]
Myzopodidae	*Myzopoda*	*aurita*	Madagascar	X			[1]
	*Myzopoda*	*schliemanni*	Province de Mahajanga	X			[2]
Molossidae	*Chaerephon*	*atsinanana*	Province de Fianarantsoa	X			[59]
	*Chaerephon*	*jobimena*	Province d’Antsiranana	X			[60]
	*Chaerephon*	*leucogaster*	Morondava				[61]
	*Chaerephon*	*pumilus*	Non-Malagasy				[62]
	*Mops*	*leucostigma*	Antananarivo	X	X		Fossils from d’Andrahomana [50]
	*Mops*	*midas*	Non-Malagasy				[63]
	*Mormopterus*	*jugularis*	Antananarivo	X	X		Fossils from Tsimanampetsotsa [51], Ankilitelo & d’Andrahomana [50]
	*Otomops*	*madagascar-iensis*	Soalala	X	X		Fossils from Ankilitelo [50]
	*Tadarida*	*fulminans*	Betsileo				[64]
Vespertilionidae	*Eptesicus*	*matroka*	Betsileo	X			[65]
	*Scotophilus*	*marovaza*	Province de Mahajanga	X			[66]
	*Scotophilus*	*robustus*	Madagascar	X			[54]
	*Scotophilus*	*tandrefana*	Parc National de Bemaraha	X			[67]
	*Pipistrellus*	*hesperidus*	Non-Malagasy				[68]
	*Pipistrellus*	*raceyi*	Province de Fianarantsoa	X			[69]
	*Hyposugo*	*anchietae*	Non-Malagasy				[70]
	*Neoromicia*	*malagasyensis*	Northeast of Tuléar	X			[71]
	*Neoromicia*	*melckorum*	Non-Malagasy				[72]
	*Neoromicia*	*nanus*	Non-Malagasy				[73]
	*Neoromicia*	*robertsi*	Anjozorobe	X			[74]
	*Neoromicia*	*somalicus*	Non-Malagasy				[75]
	*Myotis*	*goudoti*	Madagascar	X	X		Fossils from Anjohibe Cave [24]
Miniopteridae	*Miniopterus*	*aelleni*	Province d’Antsiranana				[77]
	*Miniopterus*	*brachytragos*	Province de Mahajanga	X			[78]
	*Miniopterus*	*egeri*	Province de Toamasina	X			[79]
	*Miniopterus*	*fraterculus*	Non-Malagasy	X			[66]
	*Miniopterus*	*gleni*	Tuléar	X	X		Fossils from Ankilitelo & d’Andrahomana [50]
	*Miniopterus*	*griffithsi*	Province de Toliara	X			[77]
	*Miniopterus*	*mahafaliensis*	Province de Toliara	X			[77]
	*Miniopterus*	*majori*	Betsileo				[79]
	*Miniopterus*	*manavi*	Betsileo				79
	*Miniopterus*	*petersoni*	Province de Toliara	X			[76]
	*Miniopterus*	*sororculus*	Province de Fianarantsoa	X			[80]
**Erroneous & unconfirmed records**							
Pteropodidae	*Pteropus*	*niger*	Mascarene & Reunion Islands				Madagascar records probably erroneous
Molossidae	*Mops*	*niveiventer*	Congo				Madagascar records erroneous, represent *M. leucostigma*
	*Mormopterus*	*acetabulosus*	Mauritius				No confirmed Malagasy specimens
Vespertilionidae	*Scotophilus*	*borbonicus*	Reunion Island				No confirmed Malagasy specimens

There is no known fossil record of Myzopodidae from Madagascar. Butler [25] mentioned the presence of a humerus seemingly from a myzopodid from Olduvai Gorge in Tanzania, but he did not describe the specimen in detail, only noting that the family apparently was present on the African mainland in the early Pleistocene [5], [26]. Until now, no other fossil myzopodids have been reported. Here we document the presence of two new species of a new genus of myzopodid from the late Eocene and early Oligocene in North Africa, thus extending the geographic and temporal range of the family substantially.

Like many bat families, Myzopodidae is broadly accepted as monophyletic but while simple diagnoses exist [3], [6], [27], there is no comprehensive diagnosis or description available that includes the details of dental morphology necessary for assessing affinities of incomplete fossils. In addition to describing two new myzopodid species from the early Paleogene, we here provide a comprehensive diagnosis and description for the family including details of dental morphology. Because the new myzopodid fossils are known only from lower dentitions, most of the characters noted are based on observations from extant *Myzopoda*. However, lower premolar and molar characters can also be evaluated in the fossil myzopodids described here, and the emended diagnosis and description below reflects morphology of these taxa.

### Permissions

Permissions for collecting fossil specimens were obtained from the Egyptian Mineral Resources Authority and the Egyptian Geological Museum. All necessary permits were obtained for the described study, which complied with all relevant regulations.

### Methodological Considerations

Nomenclatural Acts: The electronic edition of this article conforms to the requirements of the amended International Code of Zoological Nomenclature, and hence the new names contained herein are available under that Code from the electronic edition of this article. This published work and the nomenclatural acts it contains have been registered in ZooBank, the online registration system for the ICZN. The ZooBank LSIDs (Life Science Identifiers) can be resolved and the associated information viewed through any standard web browser by appending the LSID to the prefix “http://zoobank.org/”. The LSID for this publication is: urn:lsid:zoobank.org:pub: urn:lsid:zoobank.org:pub:DA73AE7E-88C0-4EC8-B068-6E4FDBEA84B4. The electronic edition of this work was published in a journal with an ISSN, and has been archived and is available from the following digital repositories: PubMed Central, LOCKSS.

Dental Terminology: In order to avoid confusion, we refer to the posterior two premolars of bats by their traditionally interpreted homologies rather than those described for placental mammals by O’Leary et al. [28], thus we recognize these teeth as P3/p3 and P4/p4 rather than P4/p4 and P5/p5. However, we recognize the anterior-most premolar of bats as P1/p1, rather than the more traditional P2/p2 following the analysis of Giannini and Simmons [29].

## Results

Mammalia Linnaeus, 1758.

Chiroptera Blumenbach, 1779.

Noctilionoidea Gray, 1821.

Myzopodidae Thomas, 1904.

### Emended Family Diagnosis and Description

Extant bats referred to this family share many traits that may be diagnostic for the family as a whole. Characters visible externally include: large, non-pedicellate suction pads present on the thumb and ankle; pinna large; tragus fused to the pinna; external auditory meatus partially obscured by a fleshy, mushroom-shaped process; digit II of wing reduced to metacarpal only (phalanges absent); digit III of wing with a fully-ossified nub-like third phalanx; calcar present; tail long and extends beyond the uropatagium; females with a transverse genital opening, short clitoris, and lacking pubic nipples.

**Figure 1:**
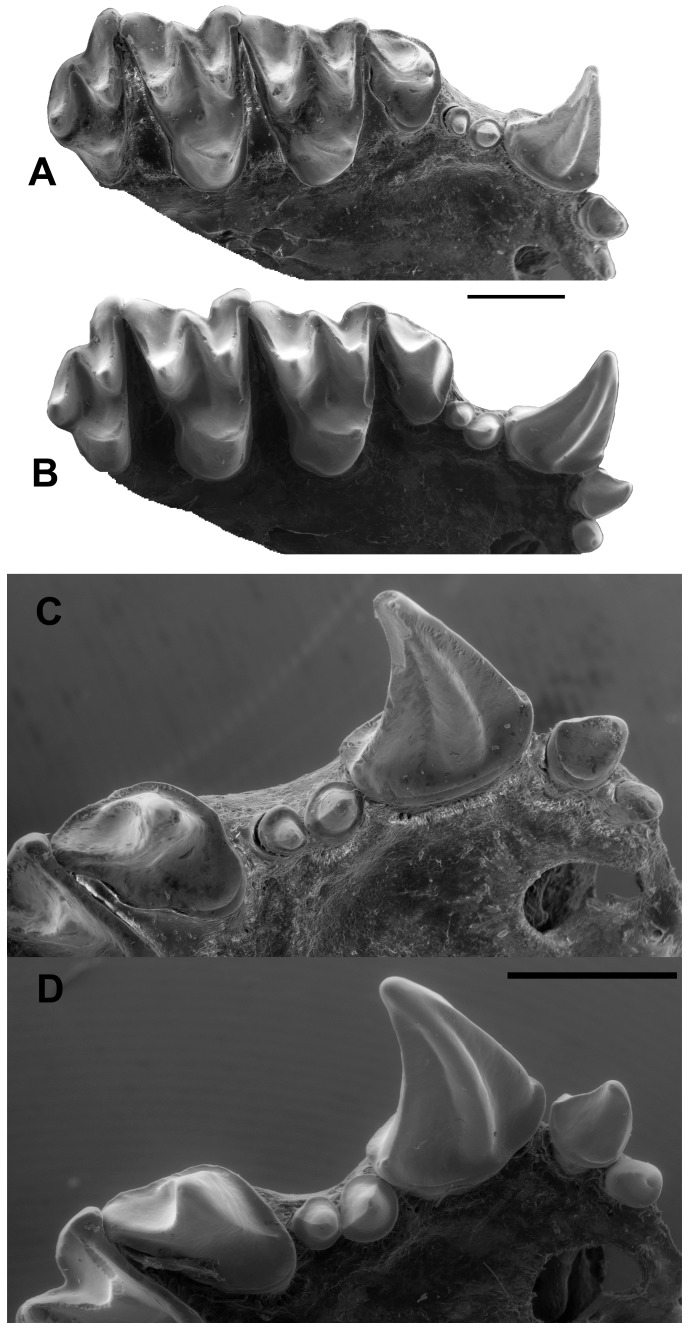
Upper dentitions of extant *Myzopoda* species. *Myzopoda aurita* (AMNH 257130), right maxillary dentition with I1-M3 in occlusal view (A) and in close-up occlusal view of right I1-P4 (C). *Myzopoda schliemanni* (AMNH 277725), right maxillary dentition with I1-M3 in occlusal view (B) and in close-up occlusal view of right I1-P4 (D). Scale bars equal 1 cm.

Skull with orthoclivous premaxilla; left and right premaxillary bodies well developed and in contact medially (Figure 1) but partially separated by a midline notch; palatine process of premaxilla complete, with lateral and medial flanges enclosing a pair of incisive foramina; nasal process of premaxilla absent; nasoincisive suture limited to a point contact between premaxilla and nasal; maxilloincisive notch absent; jugal small, does not contact lacrimal; postorbital process absent; hard palate extends posteriorly into orbital region; medial accessory palatine foramen absent; malleus with large orbicular apophysis and single tensor tympani muscle; aqueductus cochleae small or absent; epitympanic recess and stapedial fossa both shallow and broad; fenestra rotundum not enlarged; tympanic annulus inclined, does not form tubular external auditory meatus; cochlea large and phanerocochlear; stylohyal with large, ax-shaped expansion at cranial tip where it articulates with the cochlea; basihyal bar shaped and lacking an entoglossal process; angular process of dentary elongate, projects at level of occlusal plane of toothrow. Of these cranial characters, the only feature that is unique is the ax-shaped expansion of the cranial tip of the stylohyal.

Postcranial skeleton with posteriorly directed ventral accessory processes present on cervical vertebrae C2-C5; no fusion of posterior cervical or anterior thoracic vertebrae; ribs not fused to vertebrae; rib 1 not fused to manubrium; rib 2 contacts sternum at manubrium-mesosternum joint and articulates via a costal cartilage; ribs with narrow anterior lamellae and wide posterior lamellae; manubrium short, length less than 2x width, and with small anterior face; ventral process of manubrium laterally compressed to form a keel, projects at obtuse angle from body of manubrium; mesosternum narrow; xiphisternum without keel and not laterally flared; acromion process of scapula without median shelf; tip of acromion process with triangular anteromedial projection; dorsal articular facet of scapula faces dorsally and consists of a large, flat surface that is clearly separate from glenoid fossa; blade of scapula with 3 facets that are subequal in width; axillary border of scapula with a bladelike lip; anteromedial flange present on scapula; coracoid process short, stout, curved ventrolaterally, and lacking a flared tip; suprascapular process absent; clavicle articulates with scapula between acromion process and coracoid process; humerus with round head; trochiter extends beyond head; distal humerus with facets displaced from long axis of shaft; epitrochlea broad; entepicondylar foramen absent; olecranon fossa absent; ulna with reduced olecranon process; ulnar patella present.

Posterior vertebral column with no fusion of lumbar vertebrae; sacrum extends posterior to level of acetabulum; sacral lamellae broad; ascending process of ilium does not extend dorsal to iliosacral joint; dorsal ischial tuberosity absent; pubic spine present and straight, not curved dorsally; bar-like pubic symphysis present in males; distal femur shaft straight; fibula thin and threadlike; foot digits II-IV with only two phalanges on each digit; digital tendon locking mechanism absent from feet.

**Figure 2:**
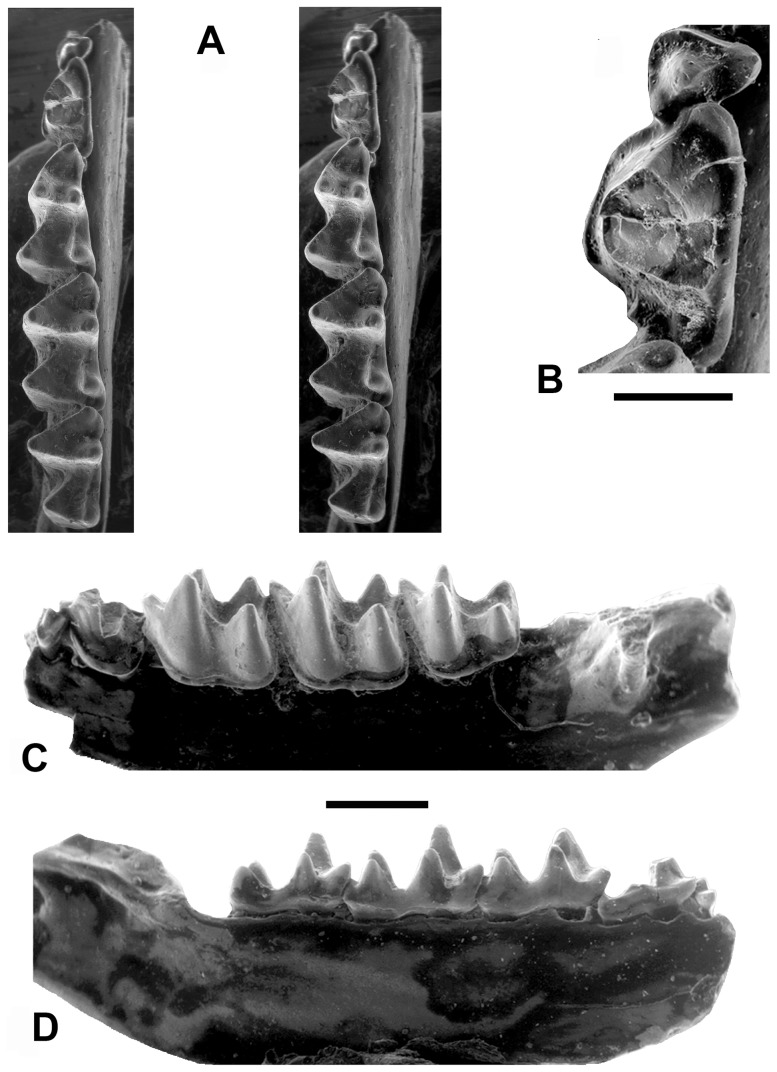
*Phasmatonycteris phiomensis*, YPM 24198, Holotype, left dentary with p3-m3. (Note: m3 talonid has been subsequently damaged, photographs taken before damage occurred). **A,** Stereophotographs of holotype in occlusal view; **B,** close-up of p3-4 in occlusal view; **C,** labial view of holotype; **D,** lingual view of holotype. Scale bar for A, C–D = 1 mm, scale bar for B = 0.50 mm.

**Figure 3:**
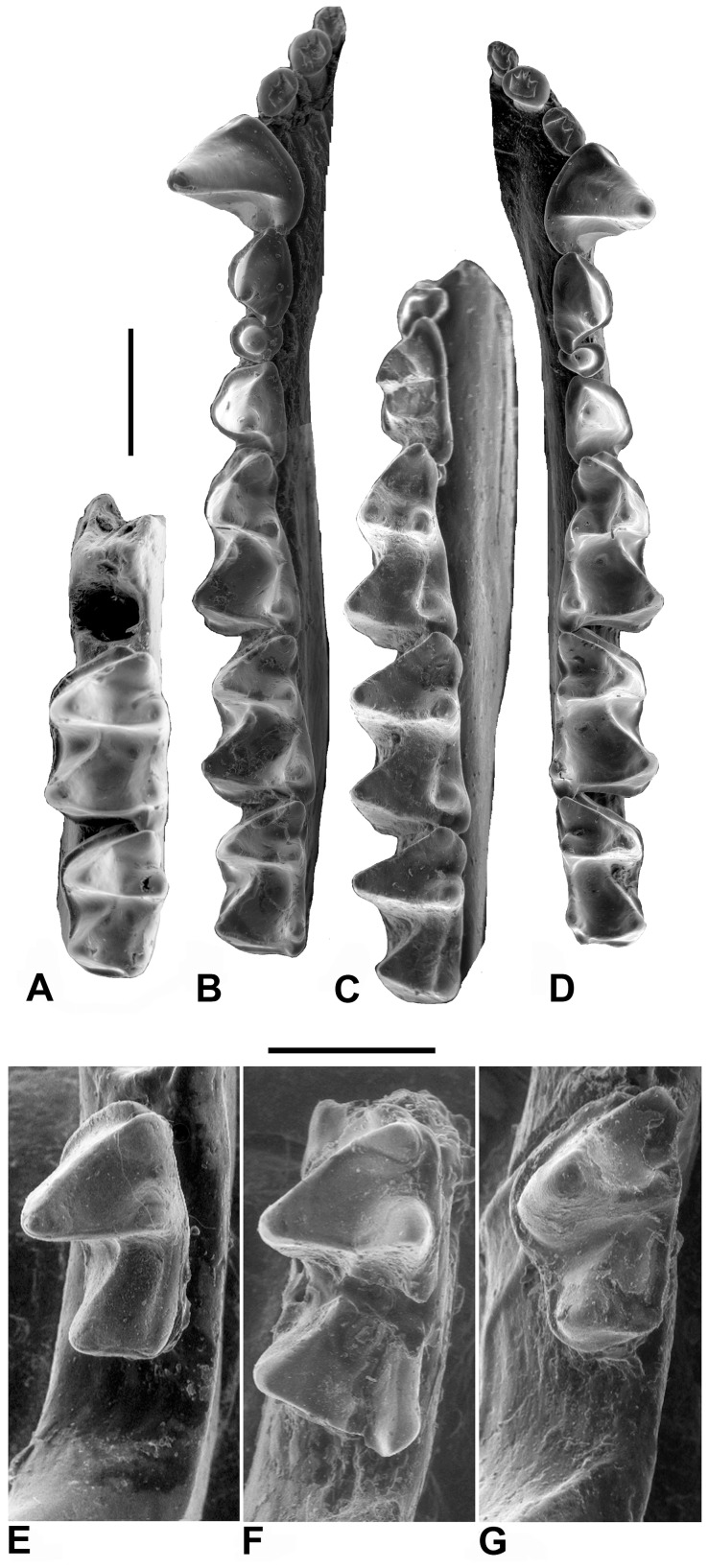
Lower dentitions of fossil and extant myzopodids. **A,** *Phasmatonycteris butleri*, CGM 83761, Holotype, right dentary (reversed) with m2-3 in occlusal view; **B,** *Myzopoda aurita*, Field Museum of Natural History (FMNH) 194176, left dentary with i1-m3 in occlusal view; **C,** *Phasmatonycteris phiomensis*, YPM 24198, Holotype, left dentary with p3-m3 in occlusal view; **D,** *Myzopoda schliemanni*, FMNH 187604, right dentary with i1-m3 in occlusal view; **E,** *P. phiomensis*, YPM 24195, left dentary with m3 in occlusal view; **F,** *P. phiomensis*, YPM 24196, left dentary with m2 (broken) in occlusal view; **G,** *P. phiomensis*, YPM 24197, left dentary with m3 in occlusal view. Scale bars = 1 mm.

Dental formula I2/3, C1/1, P3/3, M3/3 = 38 (Figs. 1–3); upper incisors proodont and retroclivous; I1 with crown clearly distinct from shaft, large main cusp that tapers to a point, and lacking a distal accessory cusp or lingual cingulum; I1 crown height less than that of I2; I1 separated from lower incisors by a large gap when jaw is in occlusion; I2 with well-developed crown including a lingual cingulum; small diastema present between I2 and C; upper C tall, height of C >5x the height of I1; upper C with complete, raised labial cingulum, no anteromedial accessory cusp, and anteromedial groove present; upper C with narrow lingual cingulum lacking a cusp or indentation; upper C with narrow distal cingulum, posteromedial ridge present, posterolingual and posterolateral surfaces flattened, posterolateral accessory cusp either absent or just a trace of cusp present; P1 single-rooted, with well-developed crown that is greater in diameter than the root, with bluntly pointed central cusp encircled by a complete cingulum on all sides; P1 either in line with P3 and P4 or slightly lingually displaced; P1 crown length longer than that of P3; P3 single-rooted, lies in line with P4 and C; P3 with well-developed cingulum surrounding central cusp and no accessory cusps; distinct diastema present between P3 and P4; P3 much smaller than P4 in all dimensions; P4 with large lingual cingulum that forms a lobe but which lacks a cusp and does not extend as far medially as protocone of M1; P4 anterior and labial cingula well developed and lacking accessory cusps; P4 with single postparacrista that decreases sharply in height and does not extend to the distal edge of the tooth; no diastema present between P4 and M1; primary cusp of P4 not in line with axis through M1 and M2 paracones, which are more lingually placed; length of P4 less than length of M1.

Upper molars tribosphenic with W-shaped ectoloph, acute angle between postparacrista and premetacrista, and lacking an ectocingulum; no diastema present between adjacent molars; M1 and M2 with protocone and paracone subequal in height, metacone taller than either of the other major cusps, mesostyle present at contact between postparacrista and premetacrista, mesostylar crest absent, paraconule and metaconule absent, parastyle present and not separated from preparacrista, single shallow ectoflexus present between parastyle and metastyle, preparacrista and postparacrista subequal in length, postmetacrista longer than premetacrista, postprotocrista oriented distolabially toward metacone but terminates before contacting the base of that cusp, hypoconal shelf and hypocone absent, endoloph absent; M3 present, reduced to 50–75% the size of M2 when seen in occlusal view, parastyle and mesostyle present, metacone developed as a distinct cusp, postparacrista shorter than premetacrista, postmetacrista absent, protocone present, hypoconal shelf and hypocone absent.

Three somewhat procumbent lower incisors present, each tooth bilobed or trilobed; no diastema present between right and left i1; i1 conspicuously smaller than i2 and i3; i2 and i3 similar in crown height; alveoli for incisors evenly spaced; small diastema present between i3 and c; right and left lower canines with bases widely separated and with laterally divergent tips; c tall and slender with subterete shaft, anterior cuspule absent, posterior cingulid present but small, posterobasal cuspule weakly developed, labial cingulid well developed and lacking cuspules; single-rooted p1 present, large and cuspidate, with distal cuspule variably present; single-rooted p3 present and in line with surrounding teeth, not offset lingually or labially; crown length of p3 much less than that of p1; p3 premolariform with a single central cusp surrounded by a complete cingulid that lacks cuspules; crown height of p3 much less than that of p4; p4 double rooted, with weakly-developed lingual cingulid that variably present, and a well-developed labial cingulid that is of roughly uniform width along the length of the tooth; p4 lacking a distinct paraconid or metaconid; p4 with tall and sharp protoconid, lingual lobe absent, talonid absent; crown length of p4 approximately 50–75% of the crown length of m1.

Lower molars tribosphenic, lacking a lingual cingulid, trigonid fovea open lingually (very broadly on m1), labial cingulid well developed and complete, labial cingulid continuous with well-developed precingulid; cristid obliqua on lower molars gently curved labially where it contacts postvallid ( = posterior wall of trigonid), lacking a notch, contacts postvallid at a point midway between metaconid and protoconid; protoconid higher than hypoconid; paraconid lower in height than metaconid; paraconid of m1 very robust, anteriorly angled, and positioned labial to line between metaconid and entoconid; metacristid, if present, markedly shorter than paracristid; entoconid well developed and tall, entocristid short, straight, and does not contact trigonid; paraconid, metaconid, and entoconid of m2–3 in alignment in occlusal view; hypoflexid shallow; m1 and m2 subequal in length, with small, low hypoconulid located on lingual edge of tooth and “twinned” with entoconid, either nyctalodont or myotodont, width of talonid basin greater than that of trigonid; double-rooted m3 with hypoconulid either present or absent, nyctalodont if hypoconulid is present; m3 crown either subequal in length to m2 or slightly shorter than m2; paraconid robust, approximately the same size as protoconid; entocristid on m3 oriented parallel to long axis of tooth; postcristid oriented either perpendicular to long axis of tooth or directed posterolabially at an oblique angle; m3 talonid either subequal to or narrower than trigonid.

The morphology of the suction pads, tragus, pinnae, and stylohyal are unique among bats and serve to distinguish Myzopodidae from all other families. One dental feature is unique to myzopodids among bats – the large, anteriorly angled and slightly labially shifted paraconid on m1. Although the paraconid is lower than the protoconid, it is of equal size and the anterior angulation broadly opens the m1 trigonid lingually and produces a steeply sloping trigonid fovea. Other dental features, while not unique to myzopodids, are distinctive of the family. The gently labially curving cristid obliqua is uncommon among bats and the complete lack of hypocones and hypocone shelves are also somewhat unusual.

*Phasmatonycteris* gen. nov. urn:lsid:zoobank.org:act:693EEE77-DD52-408B-B404-B689A43706AC.

### Generic Diagnosis

Differs from extant *Myzopoda* in having a relatively longer p4; a p3 that is more elongate and narrow, positioned labially and more closely appressed to p4; m1 and m2 of same length and m3 only slightly reduced in length; m1 with an especially robust and anteriorly angled paraconid; and all molars with more robust labial cingulids.

### Etymology

*Phasma(to)*, Greek for apparition or spectre, in reference to the long ghost lineage connecting Fayum myzopodids with extant forms, and *Nykteris*, Greek for bat.

*Phasmatonycteris phiomensis* sp. nov. urn:lsid:zoobank.org:act:94812861-7911-438E-BD80-AB849541605B (Figs. 2A–D, 3C, E–G).

### Holotype

Yale Peabody Museum **(**YPM) 24198, left dentary with p3-m3.

### Locality and Horizon

**Figure 4:**
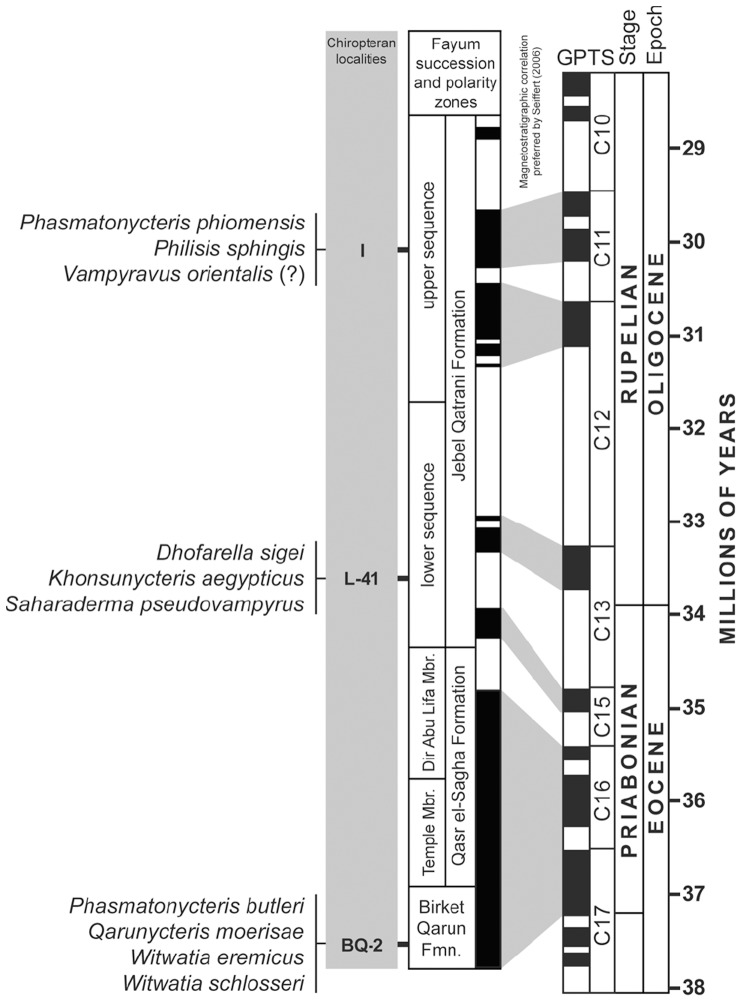
Eocene- Oligocene stratigraphy in the Fayum Depression, Egyptian Western Desert. The distribution of fossil bats from Quarries I, L-41, and BQ-2 is shown in relation to Fayum magnetostratigraphic polarity zones, epoch and stage boundaries, and correlated radioisotopic dates.

Fayum Quarry I, 242 meter level, Upper Gebel Qatrani Formation, Early Oligocene, Rupelian (∼30 Ma), Fayum Depression, Western Desert, Egypt (Fig. 4).

### Referred Specimens

YPM 24195, left dentary with m3 (Fig. 3E), YPM 24196, left dentary with m2 (3F), YPM 24197, left dentary with m3 (Fig. 3G), all from Fayum Quarry I.

### Specific Diagnosis

Differs from *P. butleri* (new species) in having sub-myotodont lower molars, a relatively larger m3 compared to m2, deeper hypoflexids on m2-3, more steeply sloping entocristids on m2-3, and m2 with a more robust, distinct, and anteriorly-oriented paraconid.

### Etymology

*Phiom*, Greek for the Fayum Region of Egypt’s Western Desert.

### Description and Comparison

The lower dentition of *P. phiomensis* is represented by four specimens – the holotype dentary preserving p3-m3, and three referred dentary fragments, two with m3 in place and the other including a broken m2. Measurements of specimens discussed in text are included in Table 2.

**Table 2:** Measurements of lower cheek teeth of extant *Myzopoda* and extinct myzopodid *Phasmatonycteris*.

Specimen	Genus/Species	p4 length	p4 width	p5 length	p5 width	m1 length	m1 tri width	m1 tal width	m2 length	m2 tri width	m2 tal width	m3 length	m3 tri width	m3 tal width
**FMNH 194176**	***Myzopoda aurita***	0.35	0.42	0.82	0.61	1.48	0.57	0.83	1.39	0.69	0.89	1.36	0.72	0.72
**FMNH 187604**	***Myzopoda schliemanni***	0.28	0.36	0.69	0.54	1.42	0.67	0.83	1.33	0.69	0.86	1.22	0.68	0.64
**YPM** **24198**	***Phasmatonycteris phiomensis***	0.39	0.41	1.17	0.68	1.64	0.67	0.86	1.58	0.89	0.94	1.38	0.91	0.67
**YPM** **24195**	***Phasmatonycteris phiomensis***											1.54	1.01	0.64
**YPM** **24196**	***Phasmatonycteris phiomensis***									0.98	0.99			
**YPM** **24197**	***Phasmatonycteris phiomensis***											1.59	1.02	0.76
**CGM** **83761**	***Phasmatonycteris butleri***								1.46	0.93	0.97	1.22	0.74	0.68

Lower p3 is dominated by a central cusp surrounded by a relatively thick, broad cingulid. The base of p3 is rounded mesiolabially with a straight margin defining the distolingual portion of the tooth (Fig. 2A–B). The cingulid is broadest mesiolingually where it forms a small shelf. It is somewhat narrower mesially, then broadens slightly and wraps around the labial border to join the distolingual portion of the cingulid. The posterior portion of p3 is positioned labial to the anterior mesiolabial margin of p5 and is appressed to the basal cingulid of p4.

The p3 morphology of *P. phiomensis* is similar to that of the p3s of extant *Myzopoda* but there are some differences. In *M. aurita* the p3 has a nearly perfectly round base and central cusp with the cingulid being slightly broader lingually (Fig. 3B). The p3 in *M. aurita* is placed directly anterior to p4 and is slightly over-lapped mesiolingually by p1. The p3 of *M. schliemanni* (Fig. 3D) has a rounded central cusp but the base is more ovoid and is wider labiolingually than it is mesiodistally long. The basal cingulid is broadest lingually and labiomesially and narrows distolingually. As in *M. aurita* the p3 of *M. schliemanni* is directly anterior to p4 and its mesiolingual portion is overlapped by p1.

The crown of the p4 of *P. phiomensis* is damaged, with the tip and the distal wall of the protoconid having been broken away (Fig. 2B). It is clear, however, that like extant *Myzopoda*, the p4 of *P. phiomensis* lacked both a paraconid and a metaconid. The p4 of *P. phiomensis* is relatively longer compared to m1 (71% of m1 length) than is the p4 in either *M. schliemanni* (49%) or *M. aurita* (55%). The crown is dominated by a relatively large protoconid that is surrounded by a basal cingulid that is slightly extended into a weak talonid shelf along the distal margin of the tooth. There is a distinct preprotocristid present that extends to the margin of the anterior cingulid as is often found in *M. aurita*. The cingulid is broadest anteriorly and narrows both labially and lingually to join the distal shelf. The anterior-most extension of the cingulid overlaps the distolingual portion of p3.

The m1 of *P. phiomensis* shares the distinctive morphology of extant *Myzopoda* (Figs. 2A, C–D, 3C). The trigonid is broadly open with a wide notch separating the paraconid and metaconid. Like extant myzopodids, the m1 paraconid is large, mesially angled and connected to the protoconid by a curving, notched paracristid. The protoconid is the tallest trigonid cusp and is positioned marginally. The metaconid is nearly as tall as the protoconid and is located almost directly lingually opposite the protoconid unlike in *M. schliemanni* and *M. aurita* where the metaconid is often slightly more distal compared to the protoconid.

The hypoconid is low and marginally placed and is connected to the trigonid by a sharply defined cristid obliqua that joins the postvallid labial of center like in extant myzopodids. The entoconid is more elevated and bulbous and is connected to the postvallid by a relatively short, straight and high entocristid. The m1 is submyotodont, unlike extant myzopodids that have fully myotodont molars. Myotodont molars have a postcristid that connects the hypoconid and entoconid, completely excluding the hypoconulid from the talonid and leaving this cusp as a small outlier that is not connected to the postcristid, but lies posterior to it. In the submyotodont condition, the postcristid extends from the hypoconid to the entoconid but there is an additional short crest that connects the postcristid to the hypoconulid as well [30]. While not common, some bat species exhibit variation in the construction of the posterior talonid. Some individuals have myotodont, submyotodont and even nyctalodont molars in the same species, and occasionally more than one condition in the same specimen [31]–[32]. In *P. phiomensis* the hypoconulid is relatively small, directly distal to the entoconid and has the postcristid either extending to it (on the nyctalodont m2) or conjoining it and the entoconid (on the submyotodont m1). There is a distinct labial cingulid present that extends around the tooth both mesially and distally.

The m2 of *P. phiomensis* (also preserved in YPM 24196, see Fig. 3F) is similar to m1 but differs by having a wider and more closed trigonid with a more labially placed paraconid and a straighter, un-notched paracristid (Fig. 2A, C–D, 3C). Like other myzopodids, the cristid obliqua turns slightly labially as it joins the postvallid.

The m3 of *P. phiomensis* (also present in YPM 24195 and YPM 24197, see Fig. 3E, G) is smaller than m1-2 but is not as relatively reduced as m3 is in extant myzopodids (Fig. 2A, C–D, 3C). The trigonid is similar to that of m2 except that it is even more closed and mesiodistally compressed. Like all myzopodids, the m3 paraconid is robust and as large as the protoconid. The m3 differs from the other lower molars by lacking a hypoconulid (extant myzopodids either have a very small m3 hypoconulid [*M. schliemanni*] or a more distinct one [*M. aurita*], see Fig. 3B, D), having a more elongate entocristid, a narrower talonid basin with the cristid obliqua joining the postvallid more lingually than in m1-2, and by having the talonid squared-off distally.

*Phasmatonycteris butleri* sp. nov. urn:lsid:zoobank.org:act:E6B5FFD8-5250-4C2A-B83F-CC469DD32557 (Fig. 3A).

### Holotype

Cairo Geological Museum (CGM) 83761, right dentary with m2-3, only known specimen.

### Locality and Horizon

Fayum Quarry BQ-2, 23 meter level, Birket Qarun Formation, Late Eocene, Priabonian (∼37 Ma), Fayum Depression, Western Desert, Egypt (Fig. 4).

### Specific Diagnosis

Differs from *P. phiomensis* in having fully myotodont lower molars, a relatively smaller m3 compared to m2, in having shallower hypoflexids on m2-3, less steeply sloping m2-3 entocristids, and m2 with a relatively smaller and less anteriorly oriented paraconid.

### Etymology

Named for Percy Butler in recognition of his work on fossil bats from Africa and of his 75 year publishing career.

### Description and Comparisons

*P. butleri* shares some distinctive myzopodid characteristics (Fig. 3A) including fully myotodont lower molars, a m2 cristid obliqua that turns labially as it joins the postvallid, a high and relatively short entocristid, and a more elongate m3 entocristid. However, in some ways this species differs from *P. phiomensis* and *Myzopoda*, especially in having less robust and less mesially angled lower molar paraconids.

The m2 of *P. butleri* is similar to that of *P. phiomensis* in having a relatively closed trigonid but the paraconid is smaller than in *P. phiomensis* and is more closely appressed to the metaconid. It also differs in having a relatively thick labial cingulid and a less robust entoconid. The m3 of *P. butleri* is a reduced version of m2 differing from the latter tooth mostly in having a narrower talonid.

## Discussion

When first described [1], *Myzopoda* was regarded as a vespertilionid, a position later supported by Dobson [33]. In 1904, Thomas proposed the family Myzopodidae for *Myzopoda* [34], recognizing the distinctiveness of the single known species at that time, *M*. *aurita*. The family was originally viewed as perhaps rather closely related to Thyropteridae because both families include species having adhesive pads on thumbs and ankles that enable them to cling to smooth surfaces such as leaves [13], [27], [34]–[35]. However, the morphological attributes of these adhesive structures are fundamentally different between the two families, suggesting that any similarities are a remarkable case of convergence [2], [13]–[15].

**Figure 5:**
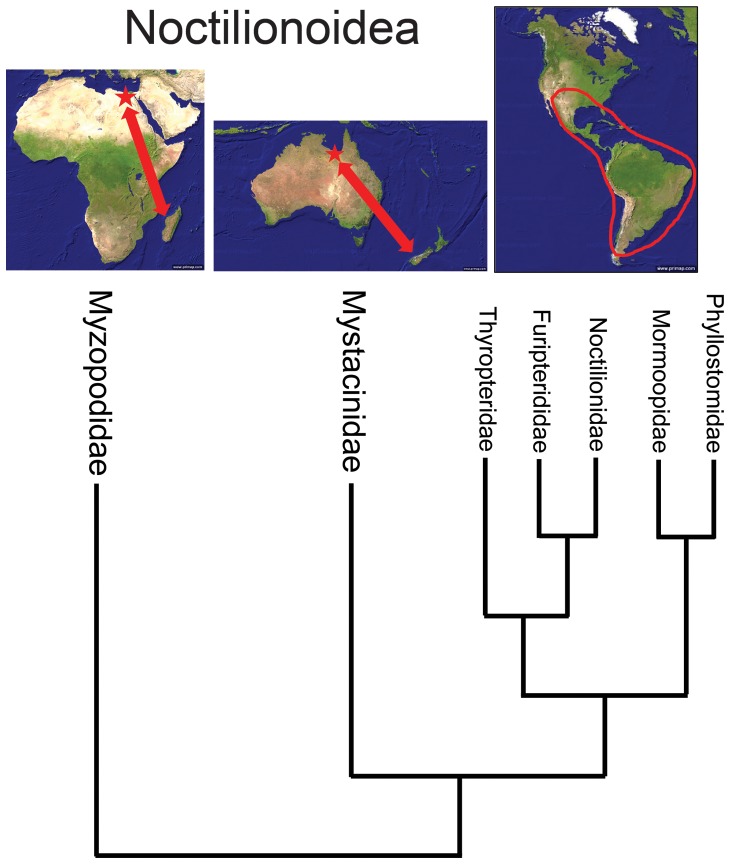
Geographic distribution of extant and fossil myzopodids and mystacinids and extant Neotropical noctilionoids. Red stars indicate location of fossil myzopodids localities (North Africa) and mystacinids localities (northern Australia) and red arrows indicate where these families are found today (myzopodids only in Madagascar and mystacinids only in New Zeeland). Extant Neotropical noctilionoids are restricted (red ellipse) to South and Central America, southern North America and the Caribbean.

Phylogenetic studies of large nuclear gene data sets indicate that Myzopodidae is most likely a basal member of the superfamily Noctilionoidea [7]–[9], a clade that also includes mystacinids, thyropterids, furipterids, noctilionids, mormoopids and phyllostomids (Fig. 5). Noctilionoidea is essentially a southern radiation [7], [9], with myzopodids restricted to Africa and Madagascar, mystacinids limited to New Zealand and Australia [36]–[37], and the other families all restricted to South and Central America and the Caribbean [38]. Teeling et al. [7] indicated that the biogeographic earliest appearances of myzopodids and mystacinids (either in Laurasia or Gondwana) were equivocal (see their figure 3) but it is now apparent that both of these families first appeared in eastern Gondwana with myzopodids being present in North Africa by the latest Eocene (∼37 Ma) and mystacinids being present in northern Australia [37] by the Oligo-Miocene boundary (∼26 Ma).

Teeling et al. [7] estimated divergence times for various bat clades using a molecular clock calibrated with several fossils. Based on their analysis, myzopodids apparently diverged from other members of Noctilionoidea between 46 and 57 million years ago. This suggests that the presence of *Phasmatonycteris* in North Africa at 37 million years ago is not necessarily a surprising occurrence. Given that other Malagasy bats (Table 1) and endemic mammals likely can trace their origin to the African mainland in the early to late Cenozoic [17], [39] a similar expectation should exist for myzopodids as well. In fact, the presence of a 37 million year old basal noctilionoid in North Africa suggests that the origins of Noctilionoidea (Fig. 5) may well be found in eastern Gondwana with a subsequent dispersal south into Australia (mystacinids) and then westward on to South America across Antarctica (lineage leading to the five Neotropical noctilionoid families).

Evidence now available suggests that Antarctica remained connected to Australia and South America until near the end of the Eocene (35 Ma) and that ice-free corridors may have existed well into the Oligocene [40]–[45]. If true, it is probable that terrestrial dispersers would have been able to reach South America from eastern Gondwana before the onset of the development of the Antarctic Circumpolar Current that mark the final separation of the three major southern land-masses [41]–[43]. Based on the presence of other Fayum and Australian bat taxa, it has been suggested that the southern continents played a crucial role in the origin and diversification of Chiroptera [46]–[49]. The new myzopodids from Egypt lend even more support to this hypothesis.
